# Understanding H3N2 subclade K: a public health concern for the 2026 influenza season

**DOI:** 10.1097/MS9.0000000000005105

**Published:** 2026-05-14

**Authors:** Muhammad Areeb Ul Haq, Zoya Ahmed, Meerab Ali Khan, Mahnoor Fatima

**Affiliations:** a Department of Medicine, Allama Iqbal Medical College, Lahore, Pakistan; b Department of Medicine, Karachi Medical and Dental College, Karachi, Pakistan; c Department of Medicine, Jinnah Hospital, Lahore, Pakistan; d Shaheed Ziaur Rahman Medical College, Rajshahi Medical University, Bagura, Bangladesh

*Dear Editor*,

Seasonal influenza continues to pose a significant global health challenge, accounting for approximately 290 000–650 000 deaths annually, disproportionately in lower- and middle-income countries, where high population density and infrastructural limitations contribute to rapid viral spread. Among multiple influenza virus strains, the ability of influenza A to emerge as subclades through antigenic drift has been a major threat to humans^[^1^]^. Recently, a novel variant of influenza A (H3N2) has been identified worldwide. It is designated as Influenza A H3N2 subclade K (J.2.4.1 lineage). Following its introduction, it has become widely circulated during the 2025–2026 influenza season, particularly among populations in East and South Asia^[^2^]^. This variant is characterized by key amino acid substitutions in the hemagglutinin (HA) protein, which facilitate enhanced binding and red blood cell agglutination, contributing to its high transmissibility^[^3^]^.

The surveillance data from ongoing influenza studies indicate the emergence of influenza H3N2 subclade K with rapid evolutionary dynamics. The virus was initially reported in Australia in mid-July 2025. It spread rapidly across the region, leading to early detection in New Zealand before global distribution. To date, it has been identified in 34 different nations, with high prevalence in the United States, Asia, Africa, and the Middle East. The enhanced evolutionary rate, increased transmissibility, and potential immune escape mechanisms highlight the virological fitness of the novel variant relative to previous influenza A H3N2 strains^[^4^]^.

H3N2 subclade K is associated with enhanced morbidity and mortality, particularly among older adults. Immunocompromised individuals and older patients with chronic conditions are more vulnerable to severe complications, owing to its enhanced pathogenicity. The complications extend well beyond acute infections, thereby contributing to an increase in hospitalization cases. Despite antigenic drift, the conventional influenza vaccine provides moderate protection against novel strains, with a reported efficacy of approximately 30–40% in adults and 72–75% in children^[^3^]^.

However, the effectiveness of current interventions is limited by certain factors, primarily the immune imprinting phenomenon, known as original antigenic sin, in the adult population. This phenomenon involves memory B cells that recall antibodies developed in response to the initial strain of influenza rather than producing antibodies that account for antigen drift. When exposed to a drifted strain, prior exposure to the antigen can limit the breadth of the immune response to the HA epitopes associated with subclade K. Evidence suggests that the rapid circulation of the virus, antigenic novelty, and immune imprinting highlight its potential for geographic spread. These factors collectively raise concerns regarding the impact of influenza H3N2 subclade K on the 2026 influenza season, particularly among older adults and high-risk populations^[^5^]^.

The urgency of its widespread evolution underscores the need to strengthen the surveillance system by implementing real-time genomic sequencing. Early laboratory testing is required to identify and report suspected cases, ensuring timely intervention. Analysis of vaccine effectiveness data and its incorporation into national immunization campaigns are crucial to protect vulnerable populations in high-risk settings. Alongside vaccination methods, antiviral therapies like neuraminidase inhibitors (e.g., oseltamivir) and cap-dependent endonuclease inhibitors (e.g., baloxavir) are important for the management of influenza and should be included in treatment guidelines^[^6^]^. Additionally, the implementation of strain-matched vaccines and the creation of next-generation universal vaccines that target conserved viral epitopes, especially in the context of individuals with less immune competence, are critical to improving protective measures. Such coordinated measures can be essential for outbreak preparedness for the 2026 influenza season, thereby reducing the burden on the healthcare system.

This article adheres to the Transparency in the Reporting of Artificial Intelligence (TITAN) guidelines, which provide a structured framework for declaring and reporting any use of artificial intelligence in research and manuscript development^[^7^]^.

## Data Availability

Not applicable to this study. No new data were generated or analyzed in this study.
